# Health Benefits of Video Games in Adolescents and Young Adults

**DOI:** 10.1007/s40124-026-00376-y

**Published:** 2026-02-26

**Authors:** Jason M. Nagata, Sahana Nayak, William Choi, Char Potes, Alexander Heuer, Zain Memon, Jonanne Talebloo, Elizabeth J. Li, Kevin Bao, Christiane K. Helmer, Megan A. Moreno, Jason M. Lavender

**Affiliations:** 1 Department of Pediatrics, University of California, 550 16th Street, 4th Floor, Box 0503, San Francisco, CA 94143 USA; 2 Department of Pediatrics, University of Wisconsin, 500 Lincoln Dr, Madison, WI USA; 3 Military Cardiovascular Outcomes Research Program (MiCOR), Department of Medicine, Uniformed Services University of the Health Sciences, 4301 Jones Bridge Rd, Bethesda, MD 20814 USA; 4 The Metis Foundation, 84 NE Interstate 410 Loop # 325, San Antonio, TX 78216 USA

**Keywords:** Video games, Digital media, Youth, Well-being, Social connection

## Abstract

**Purpose of Review:**

Although studies of outcomes associated with video gaming in adolescence and young adulthood largely focus on potential harms, video game use may also promote certain health benefits. This review synthesizes the positive health outcomes associated with video gaming, while providing recommendations to clinicians for considering and evaluating both beneficial and adverse effects.

**Recent Findings:**

Recent evidence suggests that video gaming may strengthen the development of cognitive and executive functioning in adolescents and young adults. Moreover, video game use may also contribute positively to social connection and personal identity formation.

**Summary:**

Clinicians should engage in open, nonjudgmental dialogue with youth to better explore the context and patterns of their video gaming. The American Academy of Pediatrics’ 5 Cs framework and Family Media Plan offer evidence-based frameworks that can be referenced to facilitate thoughtful discussions about video game use.

## Introduction

Video gaming is a central aspect of modern adolescent life, with studies reporting that up to 90% of teenagers [1] and 71% of children and adolescents between 2 and 17 years old play video games [2]. Moreover, about 4 in 10 teens identify as “gamers,” with 41% reporting that they play video games daily [3]. The existing literature on correlates and outcomes of video game use during adolescence is predominantly focused on potential harms. For instance, prior studies have associated gaming with elevated aggression, externalizing behaviors, gaming addiction, and mental health concerns [4–7], as well as diminished sleep quality and shorter sleep duration [8, 9].

Beyond studies focusing on the use of video games generally, other research has focused specifically on problematic manifestations of video game use. For example, internet gaming disorder (IGD), recognized by the Diagnostic and Statistical Manual of Mental Disorders, Fifth Edition, Text Revision (DSM-5-TR), is characterized by persistent and recurrent engagement in video games that results in significant impairment or distress in several aspects of a person’s life [10] and has been shown to be associated with greater conduct problems and reduced academic achievement motivation [8, 11]. However, most adolescents who engage in video gaming do not develop IGD, and problematic outcomes are primarily confined to those with risk factors, such as high impulsivity or pre-existing mental health conditions [9, 12].

In contrast to studies focused on the potentially negative impacts of video game use, a growing body of literature has begun to explore a broader set of nuanced outcomes, including potential benefits, harm-mitigation strategies, and neutral engagement associated with video gaming in adolescence. This developmental period is associated with substantial socioemotional, cognitive, and neurobiological changes [13], potentially making adolescents more sensitive to the positive and negative effects of video games. This narrative review aims to address emerging evidence on the potential health benefits of video gaming in adolescents across psychosocial, cognitive, educational, and functional domains (Table 1). Other reviews have focused on adverse consequences [8, 11], and the findings of the present review may help initiate balanced conversations between adolescents, families, and providers that facilitate recognition and discussion of both the potential risks and benefits of video game use. Additionally, the information presented here may assist clinicians in providing personalized guidance to promote healthy use and mitigate adverse effects.Other reviews have focused on adverse

**Table 1:** Summary of health benefits of video games in adolescents and young adults

Outcomes	Benefits
Psychological Well-Being	• Can support autonomy and competence, promoting intrinsic motivation • May aid stress recovery through relaxation and emotional reset
Social Connection	• Facilitates a sense of belonging • Gaming communities help support identity development • May be particularly beneficial for those who struggle with offline connection
Cognitive Functioning	• Action video games may enhance reaction time and attentional control • Strategy video games may strengthen executive functioning
Academic Performance	• May support transferable skills such as problem-solving and critical thinking • May support motivation and self-directed learning through mastery-oriented gameplay
Educational Video Games	• Increase motivation, engagement, and learning outcomes • Support skill development across different subjects
Serious Video Games	• Designed to teach or reinforce specific skills related to health and well-being • Have shown promise in mental health treatment and rehabilitation
Physical Activity	• Active video games promote physical activity by integrating movement into gameplay • Exergaming can improve fitness-related outcomes and social cohesion • Tool for physical therapy and rehabilitation

## Psychological Well-Being

A persistent concern regarding psychological well-being is the link between violence in video games and physical aggression. Although a 2018 systematic review and meta-analysis found a significant relationship between the two, the effect size was small, suggesting that context may moderate the association [14]. The American Psychological Association notes that violence is a multidimensional social problem, pointing to the need for more nuanced and in-depth investigation about the role of video game use [15]. Emerging research has begun to examine how the effects of video games may be explained by preexisting mental illness, obsessive tendencies, self-regulation, and the type of passion for video games [16, 17]. For example, one study found that those with larger amounts of harmonious passion for video gaming (stemming from a sense of deep interest and volition) experienced greater overall satisfaction with life, whereas those with obsessive passion (compulsive and overvalued compared to other aspects of one’s life) experienced more psychological distress, with self-regulation as a major mediator of these associations [16]. Thus, when used in conjunction with proper self-regulation, video games may be beneficial to the overall well-being of an individual, fulfilling some of their basic psychological needs. Moreover, another study found that the negative psychological effects of video games may depend heavily on different variables like violence, physical activity, and social connection [17]; as such, there may be certain characteristics (e.g., an “optimal gaming profile”) that make an individual more likely to benefit from playing video games [17].

While many factors may draw people to video games, major motivations can include increased psychological well-being and mood enhancement. Some researchers have theorized that the communicative process of playing structured games enables players to access new forms of autonomy [18]. The structure and environment of video games often create greater opportunity for choice and self-expression, allowing individuals to practice autonomous decision-making and better understand how to effectively express their agency. Additionally, video games can serve as important tools for recovery from stress [19]. Psychological detachment and relaxation can help individuals create distance from stressors in their lives and bring emotions back to baseline when dysregulated [20].

Self-Determination Theory (SDT) has also been applied to understanding motivations for video game use [21]. SDT asserts that the three psychological needs of autonomy, competence, and relatedness are crucial for developing intrinsic motivation [21]. These needs allow individuals to experience control over the consequences (autonomy), building skills (competence), and connecting with other players (relatedness) [21].

There is theoretical potential for video games to be beneficial for psychological well-being with some promising results, though further research is needed to quantify this association. Previous research has mainly focused on the link between violent video games and aggression, and while there is a small influence, recent research has begun to understand the contextual factors that influence whether gaming is beneficial, harmful, or neutral.

## Social Connection

In contrast to solo gaming, which occurs without real-time interaction with other players (e.g., single-player console or personal computer (PC) games) [22, 23], social gaming includes both in-person play with individuals known face to face, outside of virtual environments (e.g., playing video games in the same room) and online play with individuals whom players may or may not know offline. Social gaming may facilitate interaction and connection with co-players, offering the opportunity to meet and engage with new people [24]. Prior research has highlighted that participation in shared activities is an important factor in the development of adolescent friendships [24]. Social gaming also creates environments similar to other computer-mediated social spaces (e.g., chat rooms, social networking websites), which aid in the development of friendships between players by not only meeting new people, but by maintaining connections and friendships with existing co-players [24]. Co-players can evolve into trusted friends and sources of advice, rather than solely collaborators for achieving in-game goals [24]. Studies have found that up to 75% of online video game players report making “good friends” within these communities, and between 40% and 70% report discussing personal topics unrelated to the game, including experiences they have not shared with offline connections [24].

On the other hand, solo video games do not provide inherent opportunities for social connection or a sense of belonging [25]. Previous literature has shown that participants report greater positive emotion as a result of social video gaming rather than solitary play [25]. Many of the most popular online multiplayer video games require both teamwork and collaboration, such as *World of Warcraft*, where game-specific fan forums have evolved into digital communities with codes of conduct and hierarchies [22]. This game may facilitate strong social experiences by allowing players to interact with each other and the game’s artificial infrastructure within a virtual world [22]. Additionally, a systematic review of 263 studies found that multiplayer gaming settings may help foster friendships and a sense of belonging through shared communication and tasks [23]. It also supports identity development by helping players understand their roles within teams, engage in repeated social interaction during cooperative or competitive play, and explore interests and self-presentation through avatars or characters; collectively, these experiences can strengthen or parallel social networks [25]. However, there is some variation in how social connection is defined and measured; thus, future work should clarify what aspects of social gaming translate into meaningful relationships, including distinctions between digital communities and co-located play (e.g., playing video games in the same room with family or friends).

Overall, social gaming allows players to form relationships and experience a sense of belonging. Forming friendships this way might also be particularly helpful for adolescents who struggle to make social connections through more traditional offline means, offering an opportunity for connections that they might otherwise lack in the ‘real-world.’

## Cognitive Functioning

Cognitive functions are mental processes important for day-to-day activities, including attention, memory, and learning [26]. Executive functions are a subset of cognitive functions that allow for higher-order mental processes, such as problem solving, adaptation to novel situations, and navigation of social interactions [27]. Development of these mental processes are important in adolescent growth, shaping decision making, learning, and behavior [28]. A growing body of evidence suggests that video gaming may be associated with beneficial aspects of cognitive functioning in children and adolescents. For example, adolescents who engage in video gaming for a moderate, consistent amount of time have been shown to exhibit greater processing speed and sustained attention compared to their non-gaming peers [29–31]. Enhanced processing speed has been correlated with improved ability to perform day-to-day tasks and a lower risk of future cognitive/mobility/mood disorders [32]. However, excessive or prolonged gaming has been associated with a plateau or even decline in cognitive performance [29], highlighting the importance of moderation. Notably, different types/genres of video games may have distinct impacts on various domains of cognitive functioning due to variations in gameplay mechanics, objectives, and demands.

### Action Video Games

Games that involve rapid decision-making and fast-paced environments, such as action-oriented video games, may facilitate development of certain forms of cognitive functioning. One study of male young-adults reported that those who engaged in video game play for at least 8–10 h per week over the past year exhibited faster reaction times and heightened sensitivity to briefly presented visual stimuli [33]. Further research suggests that such benefits in gaming may extend even to novice players [34]. In one study, non-gaming university students who were instructed to play an action video game showed significant improvements in attentional control [34]. Additionally, a meta-analysis found that young adults without prior extensive gaming experience saw benefits in visuospatial ability, attention, and processing speed after video game training [35].

### Strategy Video Games

Video games that are more focused on strategy may also provide other cognitive benefits. Strategy games in particular may hone executive skills such as cognitive flexibility, working memory, and sustained attention. Unlike action video games, strategy games are often played from a third-person perspective and typically involve managing multiple, concurrent objectives. For example, real-time strategy (RTS) games, such as *StarCraft*, require players to have the ability to adapt to dynamically changing scenarios, manage resources efficiently, and perform large-scale decision-making under time constraints [36]. In one experimental study, university students with little prior video game experience spent 40 h playing *StarCraft* over a 7-week period [36]. Those who played *StarCraft* demonstrated significant improvements in cognitive flexibility, the capacity to rapidly and simultaneously maintain, evaluate, and coordinate multiple streams of information and action, compared to a control group that played *Sims*, a less situationally demanding game [36]. Following the 7 weeks, the *StarCraft* group better performed on cognitive tests of task switching, multi-source information management, and adaptation to changing task rules [36].

### Generalization of Potential Cognitive Functioning Benefits

Another area of investigation has focused on cognitive transfer, or the extent to which possible cognitive benefits associated with video gaming generalize to other tasks or activities in day-to-day living. Evidence suggests that the generalization of such benefits to external tasks is contingent upon the degree of task similarity. For example, cognitive benefits from action video games observed via laboratory tasks may be due to overlapping attentional and perceptual demands [37]. These findings underscore the importance of examining video game effects within domain-specific cognitive frameworks rather than assuming broad generalization [29].

A recent study suggests that analyzing specific game mechanics may provide a more accurate measure of cognitive transfer in video games than category/genre classifications, such as action or strategy [38]. Cognitive transfer outcomes for action video games vary substantially across studies [38–40]. Video game features such as first-person perspective, combat interactions, control of multiple objects, passive win conditions, and high time pressure are most strongly associated with improvements in attention and perception [38–40]. Such attributes require the ability to quickly adapt to in-game variables, filter relevant information, and engage in critical thinking, skills that were improved in the video game training groups [38]. One study found that students applied these problem-solving skills to their academics, demonstrating bolstered creativity and engagement in their coursework [41]. Enhancements in higher-order cognitive functions, such as working memory, reasoning, and executive control, appear linked to mechanics involving allocentric (external) perspective, combat engagement, and single controllable object gameplay [38]. These findings highlight that even video games within the same genre can vary substantially, and different games may have distinctive cognitive impacts on adolescents. Future studies investigating the benefits of video games should consider these specific factors instead of solely relying on genre classifications.

## Academic Performance

While detriments have been the primary focus of research on academic performance in relation to video game use in youth, emerging research suggests that this relationship may be more nuanced. In particular, studies linking video game use to poor educational outcomes often focus on excessive or addictive gaming behaviors [42, 43], rather than moderated or purposeful gaming. In contrast, investigations have begun to explore possible positive impacts of video games—particularly strategic and action-based video games—on academic performance. For example, one study found that strategic games were associated with enhanced problem-solving skills, which predicted greater academic performance [44]. This highlights the potential for video games to nurture skills that may also be useful for students in the classroom. Similarly, another study found that students who play video games with engaging, difficult in-game tasks may hone their creative and critical thinking skills, potentially transferable to academic and professional contexts [41]. Intrinsically motivated students who play video games that they already enjoy and wish to master may develop transferable skills through gameplay, which can then carry over into the classroom [45].

However, further research is needed to investigate the mechanisms underlying these observed relationships. For instance, the question remains whether the creative problem-solving skills applied in and potentially enhanced through video game use are transferred to academic tasks, or whether high-achieving students are more inclined to play structured, cognitively demanding games [46, 47]. Another potential mechanism is that high-achieving students may engage in video game play as a form of self-reward. One study found that high-achieving students who spent significant time playing video games were nonetheless able to maintain strong grade point averages (GPAs) [48]. Regardless, there is growing interest in leveraging video games to enhance academic skills, including critical thinking, reading comprehension, and math skills, through interactive learning [49].

## Educational Video Games

In addition to understanding potential academic performance benefits emerging from cognitive transfer associated with video game use, gaming can also be utilized for more direct educational purposes. Gamified learning platforms represent a new way to democratize learning and education by boosting student motivation, engagement, interest, and learning outcomes [50]. A variety of new platforms exist that can aid adolescents and young adults in learning across a wide range of fields, from STEM to history.

Multiplayer collaborative programming environments may allow adolescents to develop both technical and social skills through role rotation, peer feedback, and progressively challenging projects [51]. Online typing games, such as *Nitrotype*, can help adolescents build fundamental typing skills that support future career advancement. Short, leveled drills and timed challenges provide immediate feedback and chart adolescents’ advancement with their typing skills. Leaderboard-based competition with peers incentivizes incremental achievements with immediate feedback and social comparison mechanics [52]. Typing games also promote consistent practice, which can improve typing fluency. These games can be integrated into classrooms and homes as alternatives to traditional video games and social media.

Educational video games can also accelerate language learning through gamified apps, such as *Duolingo*. Language learning apps offer self-paced learning through mini modules that extend practice beyond the classroom. With gamified features, including streaks and experience points integrated with spaced repetition, language learning apps can help learners develop a consistent practice routine, making language acquisition more attainable for adolescents [52]. However, it is important to balance streaks, advertisements, and other features with the user’s best interests in mind to minimize potential negative mental health effects from using the app.

History and civilization games also represent a growing category of educational video games. Games such as *Civilization* can bring textbooks to life by immersing adolescents in decision-rich simulations that foster interdisciplinary connections, build historical reasoning, and provide perspective [53]. Skills can then be tested with trivia and quiz platforms like *Kahoo*t, which incorporate game elements such as points, rankings, competition, and cooperation, which are increasingly emphasized in classroom settings [54]. These tools may support low-stakes retrieval practice and long-term memory retention [55]. Features like team modes, immediate feedback, and varied question types further engage students while offering teachers real-time insight into learning gaps [55].

Educational video games may also complement traditional, less-engaging forms of instruction, while providing adolescents opportunities to learn and practice vital skills [50]. In the classroom, these games may offer a variety of standard teaching practices, sustain student engagement, and supply instructors with feedback about adolescents’ learning and practice at home [56]. Educational video games may be particularly beneficial for students who may struggle with traditional instruction, such as neurodivergent adolescents and young adults [57]. They are increasingly being used to support the well-being, social skills, independence, and inclusion of students with special needs in traditional educational settings [57].

## Serious Video Games

While there is no formal definition, serious video games (SVGs) are generally considered to be video games that apply elements of play to teach or reinforce specific skill sets [58]. While a comprehensive review of SVGs in health contexts is lacking, emerging studies have focused on their application in the treatment of mental health conditions. For example, a systematic review of SVGs addressing eating disorders (which had diverse skill targets such as problem solving, inhibitory control, and emotion regulation) found that 10 of 11 included papers reported improvements in certain physical, behavioral, or psychological outcomes [59]. SVGs have also been used in rehabilitation contexts, such as improving attention span in people with traumatic brain injuries. One systematic review found an improvement in 30 out of 72 measures of attention, with 42 having no effect, one having a negative effect, and four not specifically relating to attention [60]. Notably, an FDA-approved SVG called *EndeavorRX* (as well as an over-the-counter version called *EndeavorOTC*) [61] for treating attention-deficit/hyperactivity disorder (ADHD) has also recently been developed. In a randomized controlled trial of children diagnosed with ADHD funded by the developer, playing *EndeavorRX* resulted in improved attention compared to those who received the control, which was a puzzle or a word game [62]. Additionally, a systematic review concluded that game-based digital interventions may be effective in reducing substance use and promoting positive development for adolescents [63]. Taken together, results suggest the potential promise for SVGs addressing a variety of mental health concerns, but further research and additional controlled trials will be needed to better understand the efficacy of these novel forms of interventions.

## Physical Activity

Although gaming is commonly considered in relation to sedentary behavior among youth, active video games (AVGs) and exergaming may actually promote physical activity among adolescents and young adults. Unlike traditional sedentary games, which have been associated with negative health outcomes due to prolonged inactivity [64–67], AVGs incorporate bodily movement as a core component of gameplay [68]; exergaming is a specific type of AVG designed primarily for exercise rather than entertainment [68]. In this section, the term “AVG” is used broadly to include both commercial and exercise-focused games, unless specified. Popular platforms such as the Nintendo Wii, Xbox Kinect, Sony PlayStation Move, and virtual reality (VR) systems have made fitness through gaming widely accessible. Similarly, games like *Wii Sports*, *Ring Fit Adventure*, *Dance Dance Revolution*, *Beat Saber*, and *Gorilla Tag* integrate sports simulation, rhythm, and dance into a physically interactive experience, supporting both recreational fitness and rehabilitation among players [69, 70].

The interactive and immersive nature of AVGs can transform physical activity into an enjoyable and motivating experience. Features such as game-based rewards, challenges, competition, and feedback can enhance motivation, engagement, and enjoyment of physical activity, leading to increased participation in exercise [71–73]. These benefits may be particularly valuable for individuals facing challenges with motivation or self-image. For example, a study comparing adolescents with excess body weight to peers with normal weight found that AVG participation improved self-esteem, self-efficacy, and positive expectations of exercise more effectively than sedentary video games or walking activities [74]. Several studies have also demonstrated that playing AVGs can elicit physical activity levels of moderate intensity [72, 75, 76] and have found associations with improvements in body composition, including positive effects on body mass index and body fat percentage [76, 77]. In addition to physical outcomes, exergaming may support social cohesion, as cooperative play has been shown to increase pro-social behaviors and perceived social support similar to the effects of team-based physical activities [78]. AVGs may also have important applications in physical therapy and rehabilitation. In a recent randomized controlled trial, an exergame designed to train balance coordination improved both static and dynamic balance in adolescents [79]. In another study observing the effects of AVGs combined with conventional therapy on adolescents with upper limb fractures, significant improvements in range of motion and a reduction of pain were observed [80], suggesting potential applications for injury prevention and recovery. In clinical contexts, the interactive qualities of exergaming can enhance motivation, as players can receive personal feedback and enjoyment while progressing toward recovery goals [69, 74]. Home-based AVG programs further expand access to therapy by eliminating common barriers such as transportation, cost, and time constraints, making exercise more feasible for individuals with disabilities [69]. This adaptability, along with the interactive nature of AVGs, can support rehabilitation for adolescents and young adults with conditions such as cerebral palsy, stroke, burns, spinal cord injury, and spina bifida [69, 81]. However, further research on the therapeutic use of AVGs in rehabilitation for specific health conditions is necessary, as existing studies have been predominantly small-scale or pilot trials.

## Clinical Implications

Emerging research suggests that video gaming can provide certain meaningful benefits for adolescents, particularly when used in moderation. The American Psychological Association acknowledges the importance of video gaming to support mental well-being and educational outcomes for adolescents [82]. To address the complex implications of gaming, clinicians play a key role in fostering empathetic, collaborative discussions with adolescents about responsible engagement [83]. The American Academy of Pediatrics’ 5 Cs framework–Child, Content, Calm, Crowding Out, and Communication–offers a practical structure for guiding these conversations [84]. The model encourages physicians to consider adolescents’ unique motivations for gaming (“Child”), the nature of the material accessed (“Content”), the emotional or regulatory functions of screen use (“Calm”), the displacement of other activities like sleep, physical activity, and in-person interactions (“Crowding Out”), and the transparency in communication between caregivers and adolescents about media use (“Communication”) [84].

Applied specifically to video gaming, clinicians should be aware of adolescents’ motivations to play video games and their consumption patterns, which correspond to the “Child” and “Content” of the 5 Cs. They should inquire about adolescents’ reasoning behind playing video games, whether it be social connection, identity development, or entertainment purposes. In addition, clinicians should explore the specific types of video games adolescents engage with to understand potential risks, such as exposure to violent content. For marginalized adolescents who seek community and validation through gaming, counseling should be culturally sensitive, promoting safe and private online practices while recognizing the benefits of gaming for social support.

Clinicians play a key role in guiding adolescents and families in establishing a healthy balance between online and offline activities. The “Calm” and “Crowding Out” components of the 5 Cs highlight the importance of moderating screen use, stressing that digital media such as video games should complement important offline interests rather than replace them, and that online engagement should not serve as a primary coping mechanism. While video gaming can foster community and social connectedness, clinicians should inquire about nighttime screen habits and promote healthy strategies for sleep. Interventions may include helping adolescents recognize the impact of screen use on daily life and collaboratively exploring non-digital alternatives.

The “Communication” aspect of the 5 Cs framework underscores the importance of dialogue between clinicians, families, and adolescents. Previous research suggests that pediatrician encouragement can foster increased adolescent-reported communication as well as the potential for gaming co-play with caregivers [85]. These conversations can address the nature of media content, frequency of consumption, and setting boundaries. Implementing a Family Media Plan can also allow families to establish guidelines and set expectations around digital media use to promote responsible use and balanced engagement [86] (Table 2).

**Table 2:** Family media plan components adapted to adolescent video game use

Safety and Privacy within Gaming Environments	Designated Game-Free Settings	Choosing High-Quality Content
• Avoiding disclosure of personal or sensitive information • Staying away from high-risk gaming features such as unmoderated communities and predatory monetization systems • Reviewing and adjusting privacy settings to the highest protective level • Adhering to established guidelines regarding online communication	• Maintaining game-free mealtimes to promote family interaction • Keeping bedrooms free of gaming devices • Developing a structured plan for schooltime • Determining specific days or times for video gaming • Implementing a schedule that balances homework, responsibilities, and video gaming • Avoiding early-morning video games before school • Silencing and restricting devices during family time and other offline activities	• Being intentional about choosing developmentally appropriate video games • Prioritizing games that promote creativity, healthy collaboration, learning, and positive themes • Establishing clear expectations surrounding in-game purchases and downloadable content

## Conclusion

While the potential health risks of video gaming remain a topic of concern, a growing body of evidence highlights the potential benefits of video game use for adolescents and young adults, given certain important parameters (e.g., amount of use, motivations for use, game type/genre, solo versus social, sedentary versus active, etc.). Prior studies suggest that video game engagement varies across different demographic groups, including race and gender, and future studies should investigate how these factors impact both usage and outcomes, particularly the potential benefits addressed in the current review. Much of the existing literature relies on cross-sectional and observational designs, limiting interpretations of temporality and causality. Nevertheless, video games show promise as a novel avenue for adolescents and young adults to cultivate social relationships, develop cognitive skills, promote positive educational outcomes, and even increase physical activity. Clinicians should consider thoughtful, individualized approaches when addressing the risks and benefits of video game use in adolescents. 

## Key References

- Smith ET, Basak C. A game-factors approach to cognitive benefits from video-game training: a meta-analysis. PLoS One. 2023;18:e0285925. 10.1371/journal.pone.0285925.
  - This meta-analysis raises the importance of considering different in-game features in regard to cognitive transfer of skills learned in video games.
- Alwhaibi RM, Alotaibi MS, Almutairi SF, Alkhudhayr JE, Alanazi RF, Al Jamil HF, Aygun Y. Exploring the relationship between video game engagement and creative thinking in academic environments: a cross-sectional study. Sustainability. 2024;16:9104. 10.3390/su16209104.
  - This study demonstrates how video gaming in university students may lead to the development of problem solving and creativity skills, potentially applicable in academic and professional environments.
- Jaramillo-Mediavilla L, Basantes-Andrade A, Cabezas-González M, Casillas-Martín S. Impact of gamification on motivation and academic performance: a systematic review. Education Sciences. Multidisciplinary Digital Publishing Institute; 2024;14:639. 10.3390/educsci14060639.
  - This systematic review highlights the power of gamification in improving learner’s motivation and improvement of core academic competencies.
- Moller AC, Sousa CV, Lee KJ, Alon D, Lu AS. Active video game interventions targeting physical activity behaviors: systematic review and meta-analysis. J Med Internet Res. 2023;25:e45243. 10.2196/45243.
  - This meta-analysis highlights evidence showing that active video games can increase overall physical activity, suggesting their use as a tool to promote movement.

## Data Availability

No datasets were generated or analyzed during the current study.
